# Subpopulations of extracellular vesicles from human metastatic melanoma tissue identified by quantitative proteomics after optimized isolation

**DOI:** 10.1080/20013078.2020.1722433

**Published:** 2020-02-11

**Authors:** Rossella Crescitelli, Cecilia Lässer, Su Chul Jang, Aleksander Cvjetkovic, Carina Malmhäll, Nasibeh Karimi, Johanna L. Höög, Iva Johansson, Johannes Fuchs, Annika Thorsell, Yong Song Gho, R. Olofsson Bagge, Jan Lötvall

**Affiliations:** a Krefting Research Centre, Institute of Medicine Sahlgrenska Academy at University of Gothenburg, Gothenburg, Sweden; b Department of Chemistry and Molecular Biology, Faculty of Natural Sciences, University of Gothenburg, Gothenburg, Sweden; c Department of Clinical Pathology and Genetics, Sahlgrenska University Hospital, Gothenburg, Sweden; d Proteomic Core Facility, Sahlgrenska Academy at University of Gothenburg, Gothenburg, Sweden; e Department of Life Sciences, Pohang University of Science and Technology, Pohang, Republic of Korea; f Sahlgrenska Cancer Center, Department of Surgery, Institute of Clinical Sciences, Sahlgrenska Academy, University of Gothenburg, Gothenburg, Sweden; g Department of Surgery, Sahlgrenska University Hospital, Gothenburg, Region Västra Götaland, Sweden; h Wallenberg Centre for Molecular and Translational Medicine, University of Gothenburg, Gothenburg, Sweden

**Keywords:** Extracellular vesicles, exosomes, melanoma, microvesicles, subpopulations, vesicle isolation, tissue-derived vesicles, tandem mass tag, mass spectrometry

## Abstract

The majority of extracellular vesicle (EV) studies conducted to date have been performed on cell lines with little knowledge on how well these represent the characteristics of EVs *in vivo*. The aim of this study was to establish a method to isolate and categorize subpopulations of EVs isolated directly from tumour tissue. First we established an isolation protocol for subpopulations of EVs from metastatic melanoma tissue, which included enzymatic treatment (collagenase D and DNase). Small and large EVs were isolated with differential ultracentrifugation, and these were further separated into high and low-density (HD and LD) fractions. All EV subpopulations were then analysed in depth using electron microscopy, Bioanalyzer®, nanoparticle tracking analysis, and quantitative mass spectrometry analysis. Subpopulations of EVs with distinct size, morphology, and RNA and protein cargo could be isolated from the metastatic melanoma tissue. LD EVs showed an RNA profile with the presence of 18S and 28S ribosomal subunits. In contrast, HD EVs had RNA profiles with small or no peaks for ribosomal RNA subunits. Quantitative proteomics showed that several proteins such as flotillin-1 were enriched in both large and small LD EVs, while ADAM10 were exclusively enriched in small LD EVs. In contrast, mitofilin was enriched only in the large EVs. We conclude that enzymatic treatments improve EV isolation from dense fibrotic tissue without any apparent effect on molecular or morphological characteristics. By providing a detailed categorization of several subpopulations of EVs isolated directly from tumour tissues, we might better understand the function of EVs in tumour biology and their possible use in biomarker discovery.

## Introduction

Intercellular communication is crucial to maintaining the homoeostasis of all living organisms. Extracellular vesicles (EVs) are a newly discovered category of intercellular communicators that can activate surface receptors on cells and/or transfer their cargo, such as proteins, nucleic acids and lipids, into cells [1–3], which leads to a phenotypic change in the recipient cell. EVs are classified into two subtypes – microvesicles and exosomes – based on their biogenesis and characteristics [4,5]. Microvesicles are about 100–1000 nm in diameter and are released by plasma membrane budding, whereas exosomes are about 30–200 nm in diameter and are released by the endosomal pathway through the formation of multivesicular bodies [4]. However, this dichotomization has been challenged by several recently discovered subpopulations of EVs, indicating that EV complexity is greater than previously thought [6–11].

In cancer, EVs can stimulate metastasis by increasing migration [12], invasion [13], angiogenesis [3], immune modulation [14], and cell survival [15] and thereby lead to increased tumour burden. Melanoma-derived EVs have been suggested to promote tumour metastasis, and their cargo might have diagnostic and prognostic value [16]. However, to fully understand the biological role of EVs in the tumour microenvironment and their diagnostic value, the subpopulations that are present need to be identified and analysed in depth.

EVs have been successfully isolated from body fluids such as blood, urine and saliva [17,18], but studies focusing directly on tissue-derived EVs are limited [19–21], and protocols for vesicle isolation from tissue are still under development. To identify relevant EV-based biomarkers and true EV functions, tissue-derived EVs are much preferred over those derived from cell lines because cell characteristics can be influenced by long-term cell cultivation [22], which suggests that the functions of EVs are also likely to be affected. Additionally, *in vivo* and *ex vivo* samples are much preferred because cell lines might not still be representative of the tumours they were derived from after years of culturing [23,24].

Thus, there is a major unmet need for isolating and characterizing EVs from tissues in general. We have recently published a study that identified mitochondrial inner membrane proteins in a subpopulation of melanoma tissue-derived EVs. Furthermore, these mitochondrial EVs were also shown to be present in patient plasma with an in-house ELISA system, demonstrating that these EVs could reach the circulation and be a potential biomarker [20]. The overall aim of the current study was, therefore, to determine whether there are additional subpopulations of EVs present in metastatic melanoma tumour tissues, and if so whether they can be isolated *ex vivo*. To answer these questions, we developed multiple EV-isolation protocols, optimized one of them, and used it to prepare EV isolates for further detailed characterization.

## Materials and methods

### Patient information

Tumour tissues from 27 patients with a confirmed diagnosis of stage III or IV metastatic malignant melanoma were collected from consenting patients undergoing surgical tumour resection at the Department of Surgery at Sahlgrenska University Hospital during the period from May 2014 to December 2019. The tumour material used in this study was in-transit metastases, lymph node metastases, bowel metastases and liver metastases (Supplementary Table 1 and Supplementary Figure 1). The sizes of the tumour used in the study were 0.02–24 g, with the size range of the tumours used particularly for EV isolation being 0.02–15.5 g. Ethics permission was granted by the Regional Ethical Review Board at the University of Gothenburg, Sweden (#096-12 and 995–16).

### Cell culture

The human mast cell line HMC-1 (Dr Joseph Butterfield, Mayo Clinic, Rochester, MN) was cultured in IMDM (HyClone, Logan, UT) with 1.2 mM α-thioglycerol (Sigma-Aldrich, St Louis, MO). The media was further supplemented with 10% foetal bovine serum (FBS; Sigma-Aldrich), 100 units/mL penicillin (HyClone), 100 μg/mL streptomycin (HyClone) and 2 mM L-glutamine (Sigma-Aldrich). The FBS was EV-depleted by ultracentrifugation at 118,500 × *g*_avg_ for 18 h at 4°C (Type 45 Ti rotor, k-factor 217.6, Beckman Coulter, Brea, CA) and filtered through a 0.22 µm filter before added to the media. Cells were grown at 37°C in 5% CO_2_ humidified incubator. Cells were passaged every 2–3 days and cell viability was assessed using trypan blue exclusion methods.

### Isolation of EVs from HMC-1 cells

Small and large vesicles from HMC-1 cells were isolated as described previously [5] with small modifications. Briefly, cells and debris were removed by centrifugation at 300 × *g* for 10 min, and at 2000 × *g* for 20 min. Supernatant was centrifuged at 16,500 × *g*_avg_ for 20 min and 118,000 × *g*_avg_ for 2.5 h to collect large and small vesicles, respectively (Type 45 Ti rotor, 14,500 rpm, k-factor 1279.1 and 38,800 rpm, 178.6, respectively, Beckman Coulter). Pellets were resuspended in phosphate buffered saline (PBS).

### Immunohistochemistry of the metastatic melanoma tissues

Small pieces (2–3 mm) from several tumour tissues were fixed in 4% paraformaldehyde for 24 h. After washing in PBS, they were dehydrated in 70% ethanol overnight. After formalin fixation and paraffin embedding, 5-micron thick sections were cut. One section was stained with haematoxylin eosin for histologic evaluation. Additional serial sections were immunohistologically stained. The slides were treated as following. First, 3% H_2_O_2_ was added for 5 min to neutralize endogenous peroxidase. Then, 4% of bovine albumin serum was added for 20 min to ensure that no non-specific binding of the primary antibodies occured. The primary antibodies were S-100 (clone S1-61, Santa Cruz Biotechnology, Dallas, TX), GP100 (sc-59,305, Santa Cruz Biotechnology), SOX-10 (Clone EP268, Sigma-Aldrich) and Melan-A (Clone A103, Santa Cruz Biotechnology). The slides were incubated with the primary antibodies for 1 h at room temperature at a dilution of 1:100 in 1% BSA + 0.05% Triton X-100. The ready-to-use MACH 2 Universal HRP secondary antibody was then used (Biocare, Gothenburg, Sweden). The staining was visualized using a DAB chromogen kit (Biocare). Sections were counterstained with Mayer’s HTX (Histolab, Askim, Sweden). All stained sections were photographed with a U-TV0.5XC-3 camera (Olympus, Tokyo, Japan) in conjunction with a BX46 microscope (Olympus), and images were analysed with the NDPview 2 program (Hamamatsu, Hamamatsu, Japan).

### Isolation of single cells from metastatic melanoma tissues

Single-cell suspensions were prepared from metastatic melanoma tissues using the Tumour Dissociation Kit for human samples (Miltenyi Biotec, Bergisch Gladbach, Germany) and a gentleMACS^TM^ Dissociator (Miltenyi Biotec) according to the manufacturer’s protocol. Briefly, the tumours were cut into small pieces (2–4 mm) and transferred into the gentleMACS C tubes containing the enzyme mix (Solution H: 200 µl, Solution R: 100 µl, and Solution A: 25 µl) in 4.4 ml of RPMI-1640 media (Sigma-Aldrich). Tubes were placed in the gentleMACS Dissociator followed by incubation at 37°C for 30 min under continuous rotation. This was repeated twice. Single-cell suspensions were filtered through a 70 µm filter and were then treated with red blood cell lysis solution consisting of 0.1 mM EDTA (Sigma-Aldrich) in distilled water supplemented with 0.8% NH_4_Cl (Merck Chemicals, Darmstadt, Germany) and incubated on ice for 10 min to remove erythrocytes and dead cells. Two volumes of 2% FBS in PBS were added, and cells were centrifuged at 400 × *g* for 5 min at 4°C. Cells were resuspended and counted using Trypan Blue exclusion. An aliquot of cells was used to prepare cytospins, which were further stained with Hemacolor Rapid stain (Merck Millipore, Darmstadt, Germany) according to the manufacturer’s protocol. The rest of the cells were processed for flow cytometry analysis.

### Flow cytometry analysis of cells isolated from metastatic melanoma tissues

Cells isolated from tumour tissues were incubated for 15 min at 4°C with Human IgG. Briefly, the cells were stained with viability dye (LIVE/DEAD™ Fixable Aqua Dead Cell Stain Kit, Invitrogen, Life Technologies Corp, Eugene, OR) and antibodies (all from BD Biosciences, San Jose, CA) to detect surface antigens: CD3-PerCP (Clone SK7) and CD45-APC-H7 (Clone 2D1). After incubation for 30 min at 4°C in the dark, cells were washed in wash buffer (1% FBS in PBS) followed by fixation with Cytofix (BD Biosciences) for 15 min at room temperature in the dark. Finally, cells were washed in wash buffer and analysed on a BD FACSVerse^TM^ Flow Cytometry running BD FACSSuite^TM^ software (BD Biosciences). Data were analysed with FlowJo Software (Tree Star Inc., Ashland, OR). Gating of surface markers was determined using control samples with the “fluorescence minus one” approach, i.e. controls containing all markers except the marker of interest were used to set the gates. Only live cells were analysed.

### Flow cytometry analysis of HMC-1 cells and EVs to evaluate the effects of the collagenase and DNase treatment

HMC-1 cells were pelleted at 300 × *g* for 10 min and resuspended in PBS and incubated with collagenase D (2 mg/ml, Roche, Basal, Switzerland) and DNase I (40 U/ml, Roche) or with equal volume of PBS (negative control) at 37°C for 30 min with gentle agitation. After centrifugation at 300 × *g* for 10 min to remove enzymes, cells were resuspended in 50 µl of human IgG (Sigma-Aldrich) and incubated for 15 min at room temperture (RT), before being washed twice more. Cells were incubated with PE-labelled anti-CD9 (clone M-L13), anti-CD63 (clone H5C6), anti-CD81 (clone JS-81) or the corresponding isotype control (all antibodies were from BD Biosciences) and 5 µl of the vital dye 7-Amino-Actinomycin (7-AAD) (BD Bioscience) for 40 min at RT and washed twice.

Vesicles isolated from HMC-1 cells (large and small vesicles) were incubated with anti-CD63-coated beads (Thermo Fisher Scientific) overnight at 4°C with gentle agitation (10 µg EV protein/50,000 beads/antibody). Each sample was divided in two and half of the sample was treated with collagenase D (2 mg/ml, Roche) and DNase I (40 U/ml, Roche) and the other half with equal volume of PBS (negative control). After incubation at 37°C for 30 min with gentle agitation, the bead-EV complexes were washed twice with 1% EV-depleted FBS in PBS, incubated with human IgG (Sigma-Aldrich) for 15 min at 4°C, washed twice, and incubated with the same PE antibodies as the cells (anti-CD9 (clone M-L13), anti-CD63 (clone H5C6) and anti-CD81 (clone JS-81) or the corresponding isotype control (all antibodies were from BD Biosciences, 1:20 dilution)) for 40 min at RT under agitation. The samples were washed twice before analysed.

Finally, HMC-1 cells and vesicles from HMC-1 were analysed on a BD FACSVerse^TM^ Flow Cytometry running BD FACSSuite^TM^ software (BD Biosciences) with 10,000 events being acquired. Data were analysed with FlowJo Software (Tree Star Inc.).

### Transmission electron microscopy of the tissue samples

One metastatic melanoma tumour from a lymph node was dissected followed by high-pressure freezing as described previously [25,26]. Briefly, samples were placed in 150 µm-deep membrane carriers (Leica Microsystems, Bensheim, Germany) filled with 20% BSA in PBS followed by high-pressure freezing using an EMPactI machine (Leica Microsystems). A freeze substitution cocktail was applied containing 2% uranyl acetate (from a 20% uranyl acetate stock in methanol) in dehydrated acetone for 1 h after which samples were washed two times with dehydrated acetone. The temperature was increased by 3°C per hour upto –50°C, where infiltration with increasing concentrations of HM20 (3:1, 2:1, 1:1, 1:2, 1:3 acetone:HM20) followed by three changes with pure HM20. Samples were polymerized under UV light for 48 h at –50°C.

One melanoma metastasis from the liver was dissected followed by chemical fixation. Tumour tissue was post-fixed in 2% formaldehyde, 2.5% glutaraldehyde and 0.01% sodium azide in Na cocodylate buffer followed by post-fixation in osmium tetroxide 1% and *en bloc* staining in 0.5% uranyl acetate and dehydration in increasing ethanol concentrations and embedding with epon.

From both the high-pressure frozen sample and the chemically fixed sample, thin sections (70 nm) were cut with a Leica UC6 ultramicrotome (Leica Microsystems). Sections were contrasted with 2% uranyl acetate for 4 min and lead citrate for 2 min. Electron micrographs were obtained using a digitized LEO 912AB Omega electron microscope (Carl Zeiss SMT, Mainz, Germany) at 120 kV equipped with a Veleta CCD camera (Olympus-SiS, Münster, Germany).

### Isolation of EVs from metastatic melanoma tissues

Three different protocols were used to isolate vesicles from the tumour tissues, but all were based on the same technique with only minor alterations (Supplementary Figure 2). After weighing the tumour tissue, it was gently sliced into small fragments (1–2 mm) and incubated for 30 min at 37°C either in plain RPMI-1640 media (Sigma Aldrich) (Protocols 1 and 2) or in plain RPMI-1640 media supplemented with collagenase D (2 mg/ml, Roche) and DNase I (40 U/ml, Roche) (Protocol 3). After a filtration step (70 µm), cells and tissue debris were further eliminated by centrifugation at 300 × *g* for 10 min and 2000 × *g* for 20 min. Supernatants were centrifuged at 16,500 × *g*_avg_ (14,500 rpm) for 20 min and 118,000 × *g*_avg_ (38,800 rpm) for 2.5 h to collect large and small vesicles, respectively (Type45 Ti rotor, k-factor 1279.1 and 178.6, respectively, Beckman Colter). All centrifugations were performed at 4°C. The large EV- and small EV-enriched pellets were resuspended in PBS (Protocols 1 and 3). For Protocol 2 the pellets were resuspended in PBS supplemented with collagenase D (2 mg/ml, Roche) and DNase I (40 U/ml, Roche) and re-pelleted at the same speed as they were collected previously.

EVs were always isolated from fresh tumour tissue and no tissue was ever frozen prior to EV isolation. However, large and small EVs were frozen prior to be loaded on the density gradients used below.

### Transmission electron microscopy of vesicles

Investigation of vesicles by negative staining was performed as previously described [27]. Briefly, 10 µg of vesicles was placed onto glow discharged 200-mesh formvar/carbon copper grids (Electron Microscopy Sciences, Hatfield Township, PA). After two washes in H_2_O, EVs were fixed in 2.5% glutaraldehyde. After two further washes in H_2_O, the samples were stained with 2% uranyl acetate for 1.5 min. Negative-stained samples were examined on a digitized LEO 912AB Omega electron microscope (Carl Zeiss SMT, Oberkochen, Germany) at 120 kV with a Veleta CCD camera (Olympus-SiS, Münster, Germany). For size measurements, all EVs contained within the images were measured using the IMOD program [28] across the longest axis of the vesicles. A total of 297 and 277 micrographs from eight different samples were analysed for large and small EVs, respectively. A total of 3211 and 6280 vesicles was identified and measured from large and small EV samples, respectively. After iodixanol density gradient separation, samples from two isolations were investigated by electron microscopy for size calculations. Specifically, 76 micrographs of large low-density (LD) EVs and 35 micrographs of large high-density (HD) EVs were acquired, and a total of 1574 and 106 vesicles were measured for large LD EVs and large HD EVs, respectively. For small LD EVs and small HD EVs, 62 and 50 micrographs were acquired and a total of 2450 and 184 EVs were measured, respectively.

### Particle measurement

The numbers of particles were measured using ZetaView® PMX110 (Particle Metrix, Meerbusch, Germany). Measurements were done at all 11 positions and the video quality was set to medium. The chamber temperature was automatically measured and integrated into the calculation, and the sensitivity of the camera was set to 80. Data were analysed using the ZetaView® analysis software version 8.2.30.1 with a minimum size of 5, a maximum size of 5000 and a minimum brightness of 20.

### Protein measurement

The protein concentrations of tumour tissue-derived EVs isolated by protocols 1 and 2, as well as for ELISA experiments were measured by using the Pierce BCA™ Protein Assay Kit (Thermo Fisher Scientific, Waltham, MA) according to the manufacturer’s instructions. Protein concentrations in tumour tissue-derived EVs isolated by protocol 3 were valuated using the Pierce BCA™ Protein Assay Kit (Thermo Fisher Scientific) or the Qubit (Thermo Fisher Scientific) according to the manufacturer’s protocol.

The protein concentrations of the human mast cell line HMC-1, as well as the tumour tissue pellet obtained after 300 × *g* was evaluated by using RIPA buffer (Thermo Fisher Scientific) and protein inhibitors (cOmplete™, Mini Protease Inhibitor Cocktail, Roche) and was created to be used as a control for the Western blot experiments. An aliquot of the isolated EV samples was mixed with RIPA buffer and used for the Western blot analysis. The protein concentrations of samples used for Western blot experiments (cell lysate, 300 × *g* pellet and isolated EVs) were measured by Qubit (Thermo Fisher Scientific) according to the manufacturer’s protocol.

The protein concentrations of the EVs isolated from the density gradients were measured by Qubit (Thermo Fisher Scientific) according to the manufacturer’s protocol.

### Western blot analysis

Proteins were loaded and separated on precast 4–20% polyacrylamide Mini-PROTEAN TGX gels (Bio-Rad Laboratories, Hercules, CA) and transferred to PVDF membranes (Bio-Rad Laboratories). The membranes were blocked with 5% Blotting Grade Blocker Non-Fat Dry Milk (Bio-Rad Laboratories) in TBST for 1 h and then incubated with primary antibodies at 4°C overnight. All primary antibodies were diluted in 0.5% Blotting Grade Blocker Non-Fat Dry Milk in TBST. The primary antibody used were anti-calnexin (1:1000 dilution, clone C5C9, Cell Signalling Technology, Leiden, The Netherlands), anti-flotillin-1 (1:1000 dilution, clone EPR6041, Abcam, Cambridge, UK), anti-CD63 (1:1000 dilution, clone H5C6, BD Biosciences), anti-CD9 (1:1000 dilution, clone MM2/57, Millipore, Darmstadt, Germany), anti-CD81 (1:1000 dilution, clone M38, Abcam), anti-mitofilin (1:500 dilution, clone AB-2547893, Invitrogen) and anti-ADAM10 (1:500 dilution, clone 163003 R&D System, Minneapolis, MN). To investigate the CD63, CD9, CD81 and ADAM10 expression, the separation was performed under non-reducing conditions. For the other proteins, the separation was performed under reducing conditions. The membranes were washed three times before incubation with the secondary antibody for 1 h. The secondary antibody used for anti-calnexin, anti-flottilin-1 and anti-mitofilin was anti-rabbit IgG (horseradish peroxidase conjugated, 1:5000 dilution, Harlan Sera-Lab, Loughborough, UK) and the secondary antibody used for anti-CD63, anti-CD9, anti-CD81 and anti-ADAM10 was anti-mouse IgG (horseradish peroxidase conjugated, 1:5000 dilution, Harlan Sera-Lab). Both of the secondary antibodies were diluted in 0.5% Blotting Grade Blocker Non-Fat Dry Milk in TBST. The membrane was then analysed with the SuperSignal West Femto maximum sensitivity substrate (Thermo Fisher Scientific) and a ChemiDoc Imaging System (Bio-Rad Laboratories).

### ELISA

Direct ELISA was used to detect the surface expression of several EV markers. Briefly, 500 ng of large and small EVs that were isolated by Protocol 3 and dissolved in 100 µl of PBS were added to a 96-well plate and incubated overnight at 4°C. Plates were blocked with 1% BSA/PBS for 1 h. The anti-CD81 (clone H121), anti-CD63 (clone H193) and anti-CD9 (clone C-4) antibodies were added and incubated for 2 h (all antibodies were in a 1:300 dilution and purchased from Santa Cruz Biotechnology). After washing with PBS, HRP-conjugated secondary antibody (1:2000 dilution, GE Healthcare) was added and samples were incubated for 1 h. After washing with PBS, the luminescent signal was measured with the BM Chemiluminescence ELISA Substrate (BD Biosciences, San Jose, CA).

### RNA isolation and detection

RNA was extracted from 100 µg of tumour tissue-derived vesicles using a miRCURY^TM^ RNA Isolation Kit (Exiqon, Vedbaek, Denmark) according to the manufacturer’s protocol. However, for some of the large HD EV and small HD EV samples, the yield was not enough and then RNA was isolated from 200 µl of tumour tissue-derived vesicles instead (17–40 µg of proteins). The EV RNA profiles were analysed using an Agilent 2100 Bioanalyzer® (Agilent Technologies, Santa Clara, CA) and the RNA 6000 Pico Kit. One microlitre of RNA was analysed as previously described [29].

### ExoView^TM^

EV samples were analysed with the ExoView^TM^ Plasma Tetraspanin kit and an ExoView^TM^ R100 (NanoView Biosciences, Boston, MA) according to the manufacture’s instructions. The concentration of tumour tissue-derived EVs where measured with NTA and the desired concentration (1–3*10^8^ particles in total) were diluted 1:1 using incubation solution. For the HMC-1 supernatant experiments, the cells were incubated with collagenase D (2 mg/ml) and DNase I (40 U/ml) or with equal volume of PBS (negative control) at 37°C for 30 min with gentle agitation. After centrifugation at 300 × *g* for 10 min to remove cells, 50 µl of the supernatant was mixed 1:1 with incubation solution. Thirty-five microlitre of all samples was added directly to the chip and was incubated at room temperature for 16 h. Next, the samples were subjected to immune-florescence staining using fluorescently labelled antibodies (CD9/CD63/CD81, provided in the kit), washed and scanned. The data obtained were analysed using the NanoViewer analysis software version 2.8.10.

### Iodixanol density gradient

Large and small EVs from the ultracentrifugation protocol were maintained separately and dissolved in 180 µl–1 ml PBS that was mixed with 3–3.82 ml 60% iodixanol (Optiprep, Sigma Aldrich) reaching a final volume of 4 ml and 45–57.3% iodixanol. A discontinuous iodixanol gradient (35%, 30%, 28%, 26%, 24%, 22% and 20%; 1 ml each, but 2 ml for 22%) was laid on top of the sample. Samples were ultracentrifuged at 186,000 × *g*_avg_ (38,800 rpm, SW 41 Ti, k-factor 138.0) for 16 h. Large LD EVs and small LD EVs were collected from the interface of the 20% and 22% iodixanol layers, approximately at 1.111–1.121g/cm^3^ according to Optiprep^TM^ application sheet C01 (http://www.axis-shield-density-gradient-media.com/C01.pdf). Large HD EVs and small HD EVs were collected from the interface of the 30% and 35% iodixanol layers, approximately 1.163–1.189 g/cm^3^ according to Optiprep^TM^ application sheet C01 (http://www.axis-shield-density-gradient-media.com/C01.pdf) (Figure 6). The average yield (µg EV protein/g of tumour) was 758.8, 564.9, 911.2 and 37.2 for large LD EVs, large HD EVs, small LD EVs and small HD EVs, respectively. For the three samples sent for tandem mass tag (TMT) analysis samples from one tumour was loaded per gradient. For the sample used for Western blot validation of proteins identified with TMT, samples from two tumours were loaded on one gradient.

### Study design, sample preparation and digestion for mass spectrometry

An aliquot corresponding to a total protein amount of 40 µg was used from all samples and sodium dodecyl sulfate (SDS) was added to a final concentration of 2%. A reference pool was made by taking an aliquot from all samples thus generating a representative sample included in each set.

The patient samples were divided into three TMT sets, one set for each patient (Supplementary Figure 6(a)).

The proteins were digested using the filter-aided sample preparation method [30]. Briefly, samples were reduced with dithiothreitol, transferred to 30 kDa MWCO Pall Nanosep centrifugation filters (Sigma-Aldrich), washed several times with 8M urea and digestion buffer (0.5% sodium deoxycholate (SDC) and 50 mM triethylammonium bicarbonate). Alkylation was performed using methyl methanethiosulfonate and samples were digested by addition of Pierce MS-grade trypsin (Thermo Fisher Scientific) in two steps. Peptides were collected by centrifugation. Digested peptides were labelled using TMT 11-plex isobaric mass tagging reagents (Thermo Fisher Scientific) according to the manufacturer instructions followed by removing SDC by acidification with TFA. The combined samples were fractionated with basic reversed-phase chromatography on a Dionex Ultimate 3000 UPLC system (Thermo Fisher Scientific) using a reversed-phase XBridge BEH C18 column (3.5 µm, 3.0 × 150 mm, Waters Corporation). The LC-MS analysis revealed contaminating detergents for 4 of the 20 fractions in Set 2, and these samples were purified using HiPPR Detergent Removal Resin (Thermo Fisher Scientific) according to the manufacturer instructions prior a second LC-MS analysis.

### nanoLC-MS/MS analysis and database search

Each fraction was analysed on an Orbitrap Fusion Tribrid mass spectrometer (Thermo Fisher Scientific) coupled to an nLC 1200 liquid chromatography system. Peptides were trapped on an Acclaim Pepmap 100 C18 trap column (100 μm × 2 cm, particle size 5 μm, Thermo Fischer Scientific) and separated on an in-house constructed analytical column (300 × 0.075 mm I.D.) packed with 3 μm Reprosil-Pur C18-AQ particles (Dr. Maisch HPLC GmbH, Germany) using a 90-min gradient from 7% to 80% acetonitrile in 0.2% formic acid. The Orbitrap Fusion Tribrid mass spectrometer was operated in data-dependent MultiNotch MS3 mode. The full scans were acquired at a resolution of 120,000 and the MS2 scans were performed in the ion trap using a collision energy of 35%. The 10 most intense fragment ions were selected for further fragmentation using HCD and a collision energy of 65%. The MS3 scans were acquired at a resolution of 60,000.

The data files for each set were merged for identification and relative quantification using Proteome Discoverer version 2.2 (Thermo Fisher Scientific). The search was against *Homo sapiens* Swissprot Database version November 2017 (Swiss Institute of Bioinformatics, Switzerland) using Mascot 2.5 (Matrix Science) as the search engine with a precursor mass tolerance of 5 ppm and a fragment mass tolerance of 0.5 Da. Tryptic peptides were accepted with zero missed cleavage, variable modifications of methionine oxidation and fixed cysteine alkylation, and TMT-label modifications of N-termini and lysine were selected. The reference samples were used as the denominators and for calculation of the ratios. Percolator was used for the validation of identified proteins, and the quantified proteins were filtered at 1% FDR and grouped by sharing the same sequences in order to minimize redundancy. Only peptides unique for a given protein were considered for identification of the proteins, thus excluding those common to other isoforms or proteins of the same family.

### EV protein list for Figure 8

The top 100 proteins identified in EVs from three online EV databases – EVpedia, ExoCarta and VesiclePedia [31–33] – were downloaded (December 2018). Additionally, new markers for subpopulations of EVs suggested by Kowal and colleagues [6] were added to the list. Lastly, protein argonaute 2 was also added to the list because it is debated whether it is EV-associated or not [34,35]. After duplicates were removed the list contained 139 proteins that are commonly reported to be EV-associated proteins.

### Bioinformatics and statistical analysis

Where appropriate, data are expressed as the mean and standard deviation of the mean (SD). Statistical analysis was performed by non-paired Student’s *t*-test or one-way ANOVA for multiple comparisons in GraphPad Prism 6 (GraphPad Software Inc., La Jolla, CA).

Qlucore Omics Explorer (Qlucore, Lund, Sweden) was used for the principal component analysis, multi-group comparison and unsupervised hierarchical clustering. TMT set was used as an eliminating factor.

### Data availability

The proteomics data have been submitted to Vesiclepedia [33]. We have submitted all relevant data of our experiments to the EV-TRACK knowledgebase (EV-TRACK ID: EV190108) [36].

## Results

### The cellular composition of melanoma tissue and the presence of EVs in the tumour interstitial space

**Figure 1.**
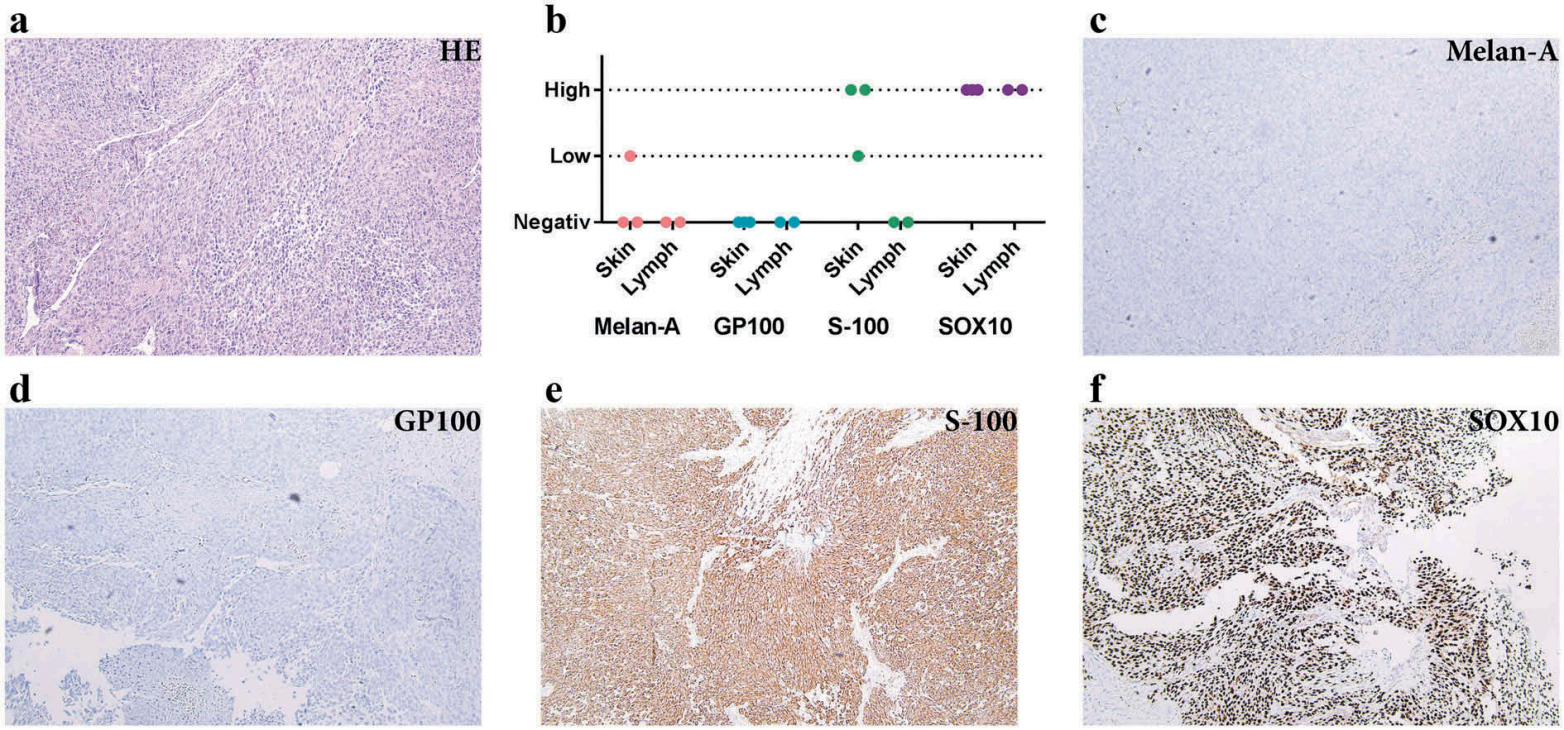
Immunohistochemistry analysis shows S-100 and SOX10 positivity in the metastatic melanoma tumour. (a) Haematoxylin and eosin staining of metastatic melanoma from lymph nodes composed of atypical pleomorphic elongated melanocytes with high-mitotic activity and focal necrosis. (b) Expression of melanocytic markers in metastatic melanoma from skin and lymph nodes (immunohistochemistry). (c–f) Immunohistochemical profile of a representative tumour: Melan A negative (c), GP100 negative (d), S-100 positive (e) and SOX10 positive (f). All images are at 100 × magnification. *N* = 5 for all stainings.

First, we determined the cellular composition of metastatic melanoma tissues. The tumour tissues had small areas of necrosis but consisted mostly of atypical spindle-shaped melanocytes as shown by histologic examination (Figure 1(a)). Routine immunohistochemical staining for known markers of melanoma showed higher expression of S-100 protein and SOX-10 than Melan-A and GP100 (Figure 1(b-f)), which is consistent with previous observations [37–40].

**Figure 2.**
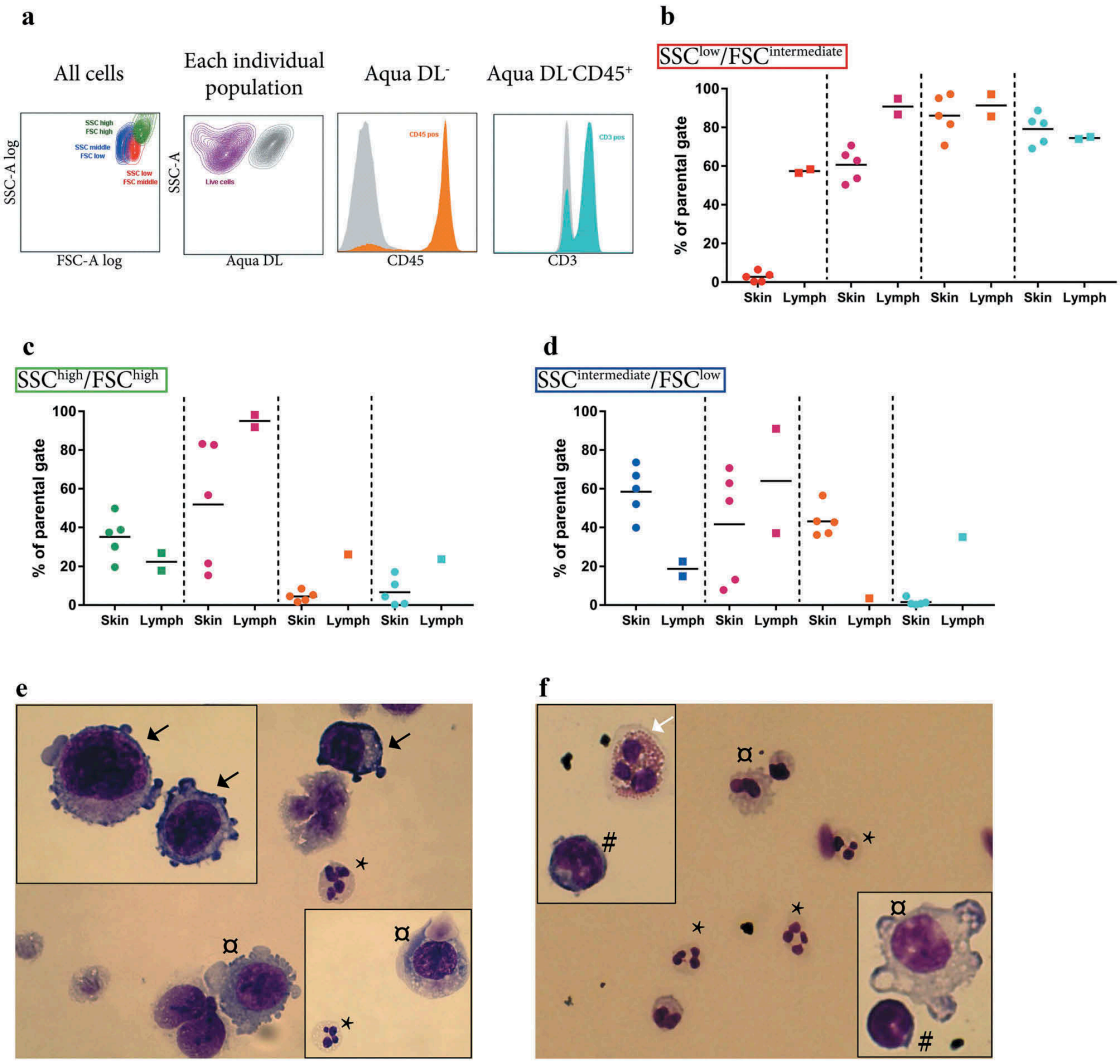
Analysis of the cellular composition of tumour tissues by flow cytometry. (a) Flow cytometry showed that the majority of the tumours consisted of three distinct populations in SSC-A log and FSC-A log, namely an SSC^low^/FSC^intermediate^ population (red, further analysed in b), an SSC^high^/FSC^high^ population containing the largest and the most granulated cells (green, further analysed in c), and an SSC^intermediate^/FSC^low^ population (blue, further analysed in d). (b–d) Percentages of live cells (purple), cells positive for the leukocyte marker CD45 (orange), and CD45^+^ cells also positive for the T lymphocyte marker CD3 (turquoise) are shown separately for the SSC^low^/FSC^intermediate^ (b), the SSC^high^/FSC^high^ (**c**), and the SSC^intermediate^/FSC^low^ population (d). *N* = 5. (e–f) Hemacolor Rapid staining of the single cell-suspension of subcutaneous melanoma metastasis (e) and lymph node metastasis (f). Black arrows, tumour cells; white arrows, eosinophils; *, neutrophils; #, lymphocytes; ¤, macrophages. *N* = 5.

The majority of the tumours had three distinct populations of cells as determined by flow cytometry (Figure 2(a)). In lymph node metastases, lymphocytes (CD45^+^CD3^+^) were the most abundant cell type, whereas in skin metastases small, non-lymphoid immune cells (CD45^+^CD3^−^, possibly neutrophils) were most abundant (Figure 2(b,d)). Large, granulated cells were common in both lymph node and skin metastases (Figure 2(c)). Most of these large cells were CD45^−^ and thus most likely to be tumour cells. However, a few cells were CD45^+^CD3^−^ and these might represent macrophages. In a separate analysis, Hemacolor Rapid staining confirmed the presence of neutrophils, macrophages, lymphocytes and eosinophils as well as melanocytes in the single-cell suspensions (Figure 2(e-f)).

**Figure 3.**
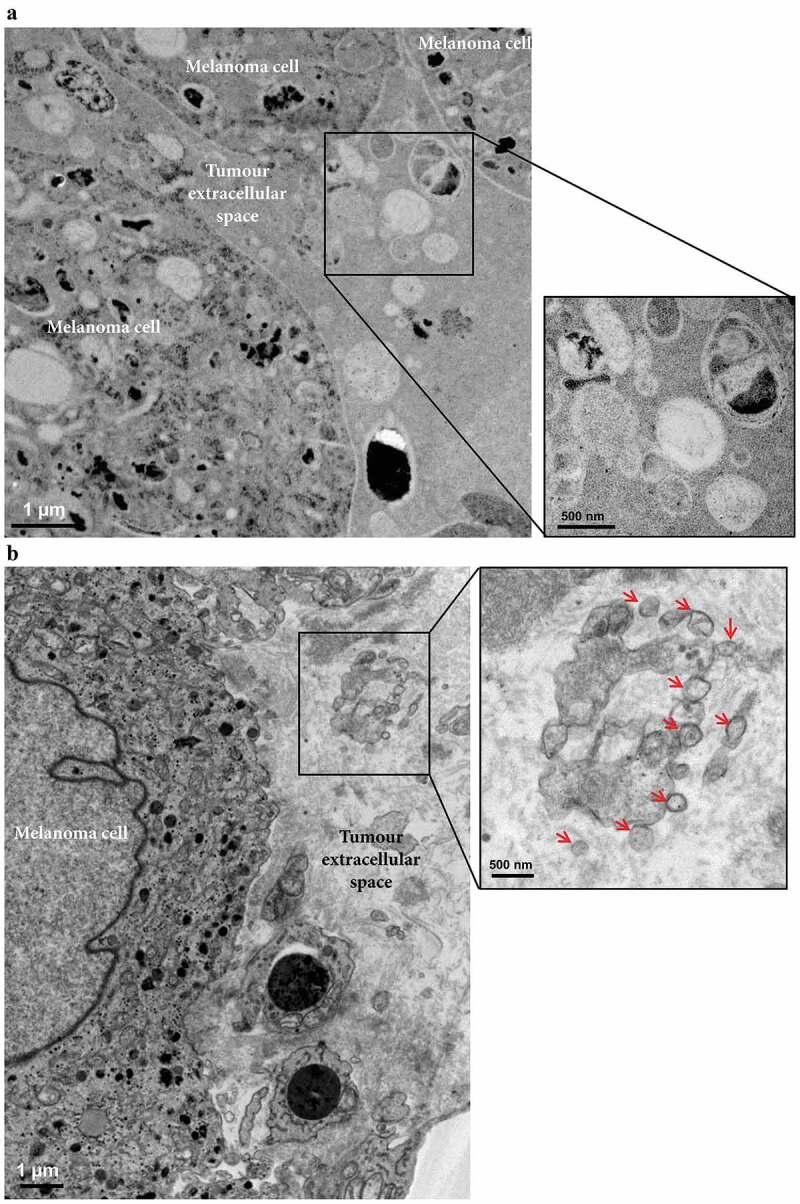
Electron microscopy analysis of extracellular vesicles in metastatic melanoma tissues. (a) High-pressure frozen melanoma thin section. Melanin accumulation in the cells is clearly evident. The higher magnification image shows different types of vesicles in the extracellular space. *N* = 1. (b) Melanoma thin section prepared by chemical fixation. A large tumour cell and two lymphocytes are shown. Black stain, possibly melanin, is clearly visible inside the melanoma cells, and the melanoma cells are well recognized by their cell membrane. The higher magnification image shows EVs (red arrows) in the extracellular space. *N* = 2.

Next, we determined the presence of vesicles within the melanoma metastatic microenvironment by electron microscopy (Figure 3(a,b)). Black structures possibly corresponding to Melanin were clearly visible inside the melanoma cells, and additional small cells, most likely lymphocytes, were also present. Importantly, vesicular structures of various sizes, with similar morphologies as EVs were seen in the melanoma interstitial space, similar to what has been previously observed in prostate cancer tissues [41]. Together these results demonstrate that the tumour microenvironment consists of both tumour cells and immune cells and that the interstitial space harbours EVs.

### Establishment of the EV isolation protocol and characterization of the isolated EVs

**Figure 4.**
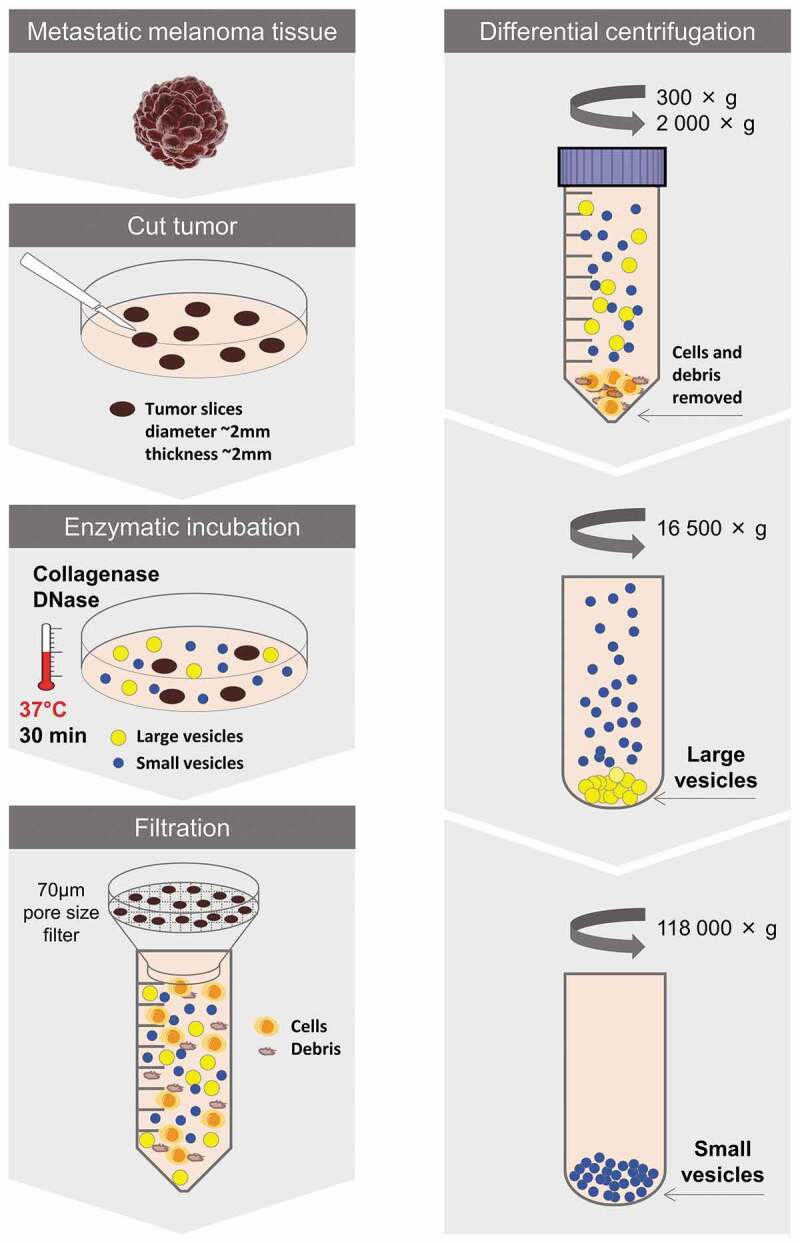
Schematic overview of the centrifugation-based protocol used to isolate vesicles from metastatic melanoma tissues. The tumour tissues were dissected into smaller pieces that were incubated in cell culture media containing collagenase D and DNase I for 30 min allowing the tissue EVs to be released into the media. EVs were isolated from the media by differential centrifugation resulting in two subpopulations of EVs (large EVs (16,500 × *g*_avg_ pellet) and small EVs (118,000 × *g*_avg_ pellet)).

To be able to further study these vesicular structures, we next sought to develop a protocol for isolation of EVs directly from tumour tissues. One protocol was established that first involved the careful dissection of the tumour to allow for a greater tissue surface to be accessible. Second, the tumour pieces were incubated in cell culture media for 30 min allowing the tissue EVs to diffuse into the media. Third, EVs were isolated from the media by differential ultracentrifugation resulting in two subpopulations hereafter termed large EVs (pelleted at 16,500 × *g*_avg_) and small EVs (pelleted at 118,000 × *g*_avg_) (Protocol 1, Supplementary Figure 2). However, the vesicle-enriched pellets that were obtained were difficult to resuspend and tended to create aggregates, so we added an enzymatic treatment step – DNase I and collagenase D – to the pellets for 30 min (Protocol 2, Supplementary Figure 2). Although we could isolate vesicles with both of these protocols (Supplementary Figure 3(a-b)), the data suggested that vesicles were lost in the process because a re-pelleting step was required (Supplementary Figure 3(c-d)). The best yield was instead observed when DNase I and collagenase D treatment was performed on the tissue pieces during the 30 min of incubation in culture media prior to EV isolation (Protocol 3, Supplementary Figure 2) and we therefore continued with Protocol 3 for the subsequent experiments (Figure 4). It has previously been shown that collagenase do not affect the expression of surface molecules on cells [42]. To test this on EVs we evaluate the effect of our collagenase D and DNase I cocktail on a cell line, HMC-1, as we did not have enough material to test it on the tissue-derived EVs. When the HMC-1 cells were treated no effect on the viability was seen (95% and 96% in control and enzyme-treated cells, respectively, as determined with Trypan blue). Furthermore, no effect was seen on the expression of CD9 and CD63 on the cells, nor on the EVs analysed with Flow cytometry and ExoView^TM^ (Supplementary Figure 4(a-c)). However, we saw a small reduction of CD81 expression on both cells and EVs (Supplementary Figure 4(a-c)).

**Figure 5.**
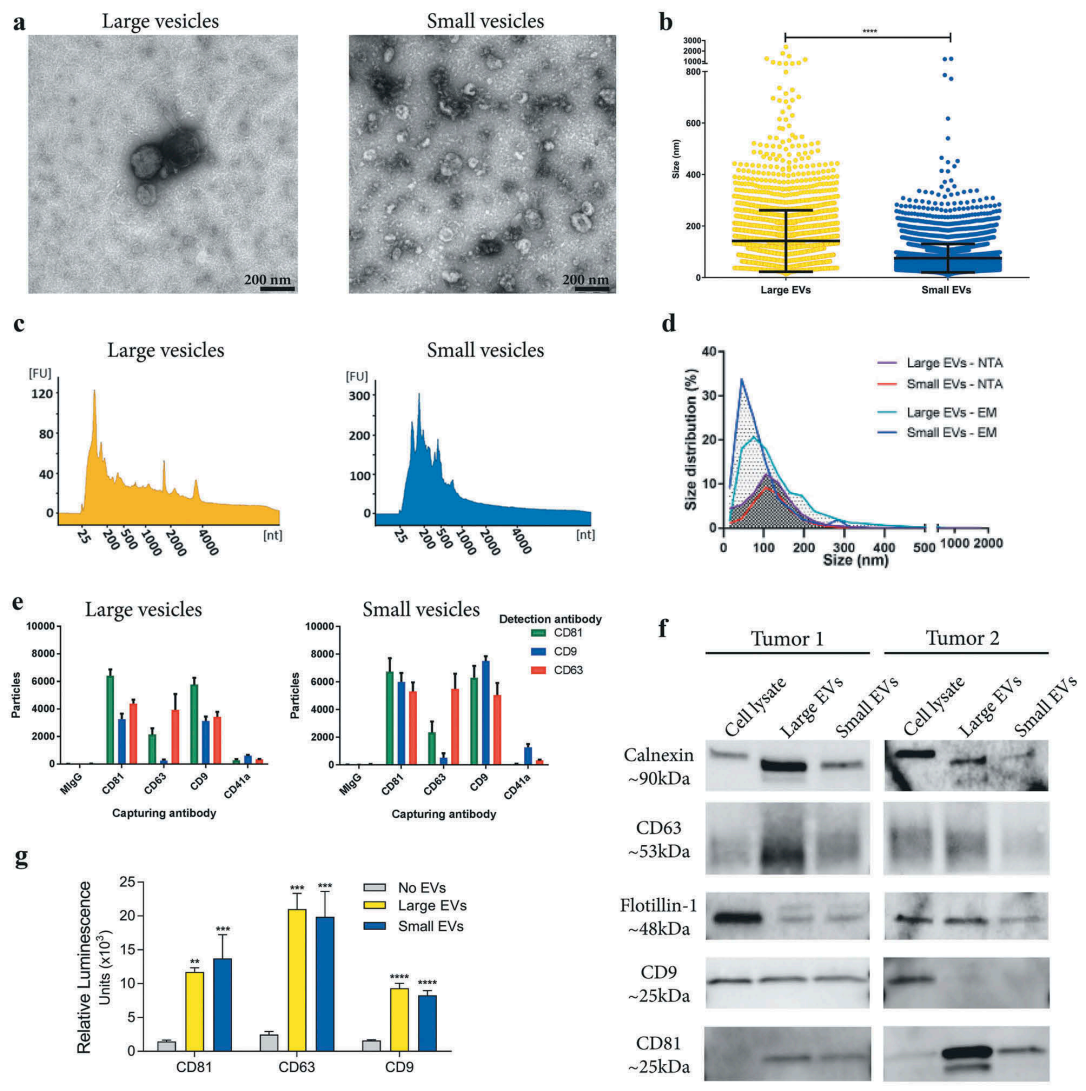
Characterization of large and small EVs isolated from metastatic melanoma tissues. (a) Ten micrograms of large and small EVs were loaded onto grids, negative stained, and evaluated with transmission electron microscopy. Scale bars are 200 nm. (b) All structures in the 297 micrographs of large EVs and in the 277 micrographs of small EVs were measured. In total, the diameters of 3211 and 6280 structures were measured for large and small EVs, respectively. ****p-Value < 0.001; *N* = 8. (c) RNA was isolated with a miRCURY RNA Isolation Kit – Cell and Plant (Exiqon) directly from the EV pellets and was analysed with a Bioanalyzer® (Agilent). *N* = 10. (d) Size distribution obtained using nanoparticle tracking analysis (NTA; ZetaView®, *N* = 8) and the manual calculation from the transmission electron microscope. Size distribution is presented as graphs with the percentage of structures on the *y*-axis and the diameter of the structures in nanometres on the *x*-axis. (e) ExoView^TM^ showing the presence of CD9, CD63, CD81 and CD41a on the large and small EVs. The results are presented as the average ± SD from three different spots on the chip (*N* = 1). Concentration of vesicles was determined with NTA and 1*10^8^ were added to the chips. (f) Western blot was used to investigate the presence of the classical vesicle markers flotillin-1, CD63, CD9 and CD81, as well as the ER protein, calnexin. A total of 10 µg were loaded per sample. *N* = 2. (f) Direct ELISA showing the expression of CD9, CD63 and CD81 on the large and small EVs (*N* = 3). The results are presented as the average ± SD. ****p-Value < 0.0001; ***p-Value < 0.001; **p-Value < 0.01.

Tissue-derived large and small EVs showed typical morphologies and a clear difference in size (mean size 142 nm vs. 75 nm, respectively) when examined by electron microscopy (Figure 5(a-b) and Supplementary Figure 5(a)), whereas nanoparticle tracking analysis (NTA) was unable to show a difference in size between the populations (mean size 126 nm vs. 122 nm, respectively) (Figure 5(d)). Furthermore, the large vesicles contained small RNAs as well as visible peaks for the 18S and 28S ribosomal RNA as determined by high-resolution electrophoresis (Bioanalyzer®) (Figure 5(c)). In contrast, small vesicles had a broader peak for small RNAs, but no prominent ribosomal RNA peaks (Figure 5(c)), which confirmed our previous findings in cell culture-derived large and small EVs [5,43]. Further, the presence of several classical EV markers was determined by Western blot, ExoView^TM^ and a direct ELISA system. To different degrees, both large and small EVs were positive for calnexin, a marker of the endoplasmic reticulum, as well as for the classical EV proteins, flotillin-1, CD63 and CD81 as determined by Western blot. Only vesicles from one out of two the tumours used for Western blot were positive for CD9 (Figure 5(f)). Additionally, the ELISA assay and the ExoView^TM^ confirmed that both large and small vesicles were positive for the tetraspanins CD81, CD63 and CD9 (Figure 5(e,g)). Furthermore, ExoView^TM^ showed only minor positivity for the platelet marker, CD41a, on both large and small EVs, indicating low contamination of blood-derived EVs. Together our results show that EVs can be isolated directly from tumour tissues and, importantly, that enzymatic treatment that reduces aggregation of vesicles does not affect their molecular characteristics or morphologies.

### Identification of subpopulations of EVs based on density

**Figure 6.**
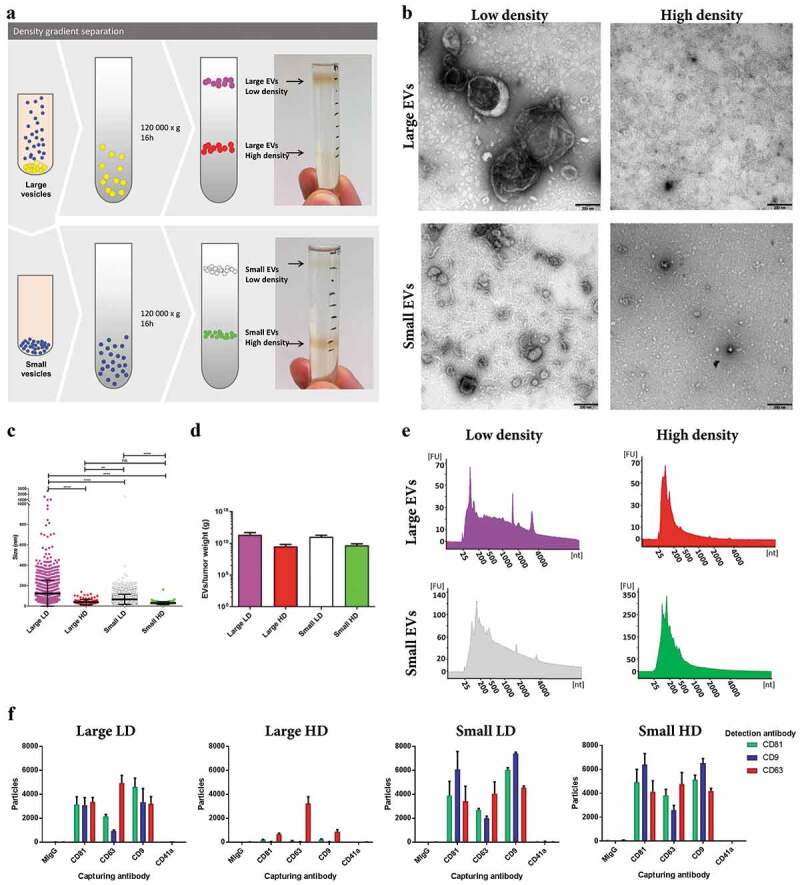
Characterization of HD EVs and LD EVs isolated from metastatic melanoma tissues. (a) Schematic overview of the vesicles isolated through a density gradient separation. Isolated large and small EVs were bottom loaded on an iodixanol gradient (45–20%). From both populations of vesicles, two fractions were visible after the separation, with one fraction containing HD EVs (1.16–19g/cm^3^) and one containing LD EVs (1.11–1.12g/cm^3^). (b) Ten micrograms of LD and HD vesicles from both large and small EVs were loaded onto grids, negative stained, and evaluated by transmission electron microscopy. Scale bars are 200 nm. (c) All structures in the large LD EV fraction (76 micrographs; 1577 vesicles), large HD EV fraction (35 micrographs; 106 vesicles), small LD EV fraction (62 micrographs; 2450 vesicles) and small HD EV fraction (50 micrographs; 184 vesicles) were measured. ****p-Value < 0.001; **p-Value = 0.008; ns = not significant; *N* = 2. (d) The concentration of EVs was determined in all samples with NTA (ZetaView®), *N* = 3, and the results are presented as the mean ± SD. (e) RNA was isolated with a miRCURY RNA Isolation Kit – Cell and Plant (Exiqon) directly from the HD and LD fractions of the density gradients and was analysed with a Bioanalyzer® (Agilent). *N* = 3. (f) ExoView^TM^ showing the presence of CD9, CD63, CD81 and CD41a on large LD and HD EVs and on small LD and HD EVs. The results are presented as the average ± SD from three different spots on the chip (*N* = 1). Concentration of vesicles was determined with NTA and 2*10^8^ (large LD EVs), 3*10^8^ (large HD and small LD EVs) and 1*10^8^ (small HD EVs) were added to the chips.

To further separate EV subpopulations, the large and small EVs were loaded on individual iodixanol density gradients. Two visible bands were observed for both subpopulations of EVs at approximately 1.116 g/cm^3^ and 1.176 g/cm^3^, hereafter termed LD and HD EVs, respectively (Figure 6(a)). As for the large and small EVs, the four new subpopulations of EVs were visualized and characterized by electron microscopy, Bioanalyzer®, and NTA. The large LD vesicles were significantly larger in size than the small LD vesicles as determined by electron microscopy (Figure 6(b-c)). However, it was also shown that the HD vesicles from both large and small EVs were the smallest in size, as well as in numbers (Figure 6(b-d)). The LD subpopulations from both the large and small EVs had small peaks for ribosomal RNA subunits, while the HD EVs did not (Figure 6(e)), indicating that the EVs associated with ribosomal RNA are of lower densities, which confirms our and others’ previous findings in cell lines [7,9,30,34]. ExoView^TM^ confirmed that all subpopulations contained CD81, CD63 and CD9 positive vesicles, however, the large HD vesicles where mainly positive for CD63 (Figure 6(f)).

### Differentially expressed proteins in subpopulation of tumour tissue-derived EVs revealed by quantitative proteomics

**Figure 7.**
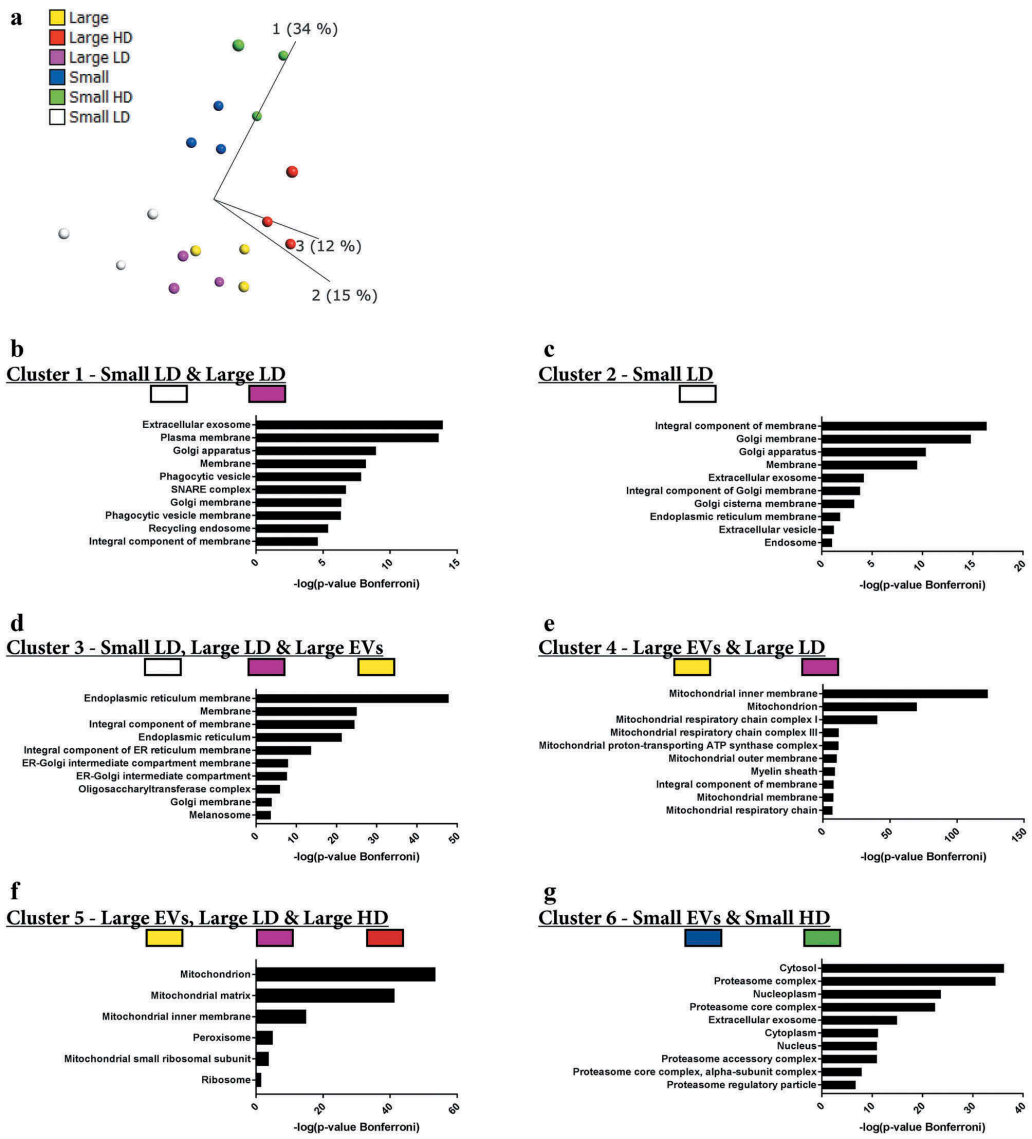
Quantitative mass spectrometry analysis of EV subpopulations from metastatic melanoma tissues. Quantitative proteomics (TMT) was used to determine the differences in the six EV isolates, *N* = 3. (a) Principle component analysis illustrating the relationship between large EVs (yellow), large HD EVs (red), large LD EVs (purple), small EVs (blue), small HD EVs (green), and small LD EVs (white). (b–g) DAVID was used to determine the most enriched cellular compartments associated with the proteins in the six clusters from the multi-group comparison (742 proteins, *p*-value = 0.001, *q*-value = 0.009), as shown in Supplementary Figure 7. The 10 most enriched terms (based on *p*-value) in each category are displayed.

Next, we performed quantitative mass spectrometry on the six types of EV samples that we had isolated so far – large EVs, small EVs, large HD EVs, large LD EVs, small HD EVs and small LD EVs – all analysed in biological triplicates (Supplementary Figure 6(a)). A total of 6870 proteins were identified, with 6832 proteins also being quantified with TMT analysis. Of these, 4514 proteins were quantified in all three TMT sets (Supplementary Figure 6(b)). To visualize the relationship between the different types of isolated EVs, a principle component analysis was performed including all quantified proteins. Component 2, representing 15% of the variability, distinguished the three samples with small EVs from the three samples with large EVs (Figure 7(a)). However, component 1, representing the largest variability with 34%, distinguished the HD samples from the LD samples (Figure 7(a)), indicating that the proteomes of the subpopulations of EVs were more similar for EVs of similar density than for EVs of the same size.

A multi-group comparison showed that 742 proteins were differentially expressed among the samples (*p*-value = 0.001, *q*-value = 0.009211), and when an unsupervised hierarchical clustering was performed these proteins were divided into six large clusters (Supplementary Figure 7). Clusters 1 and 2 contained proteins that were mainly enriched in the small LD EVs and to a lesser extent in the large LD EVs. Most of the proteins in clusters 1 and 2 were membrane proteins and were associated with the GO terms “Extracellular exosome”, “Plasma membrane”, “Endosome”/“Recycling endosome” and “Golgi apparatus” (Figure 7(b-c)). In contrast, clusters 3, 4 and 5 contained proteins enriched in several of the large EVs samples, and these proteins were associated with GO terms connected to the “Endoplasmic reticulum” or “Mitochondrion” (Figure 7(d-f)). Cluster 6 was the only cluster containing proteins enriched in both the small EVs and small HD EVs. This cluster contained proteins that were associated with “Cytosol”, “Proteasome” and “Nucleoplasm” (Figure 7(g)). Additionally, one protein – DnaJ homolog subfamily C member 13 (also called RME-8) – did not match to any of the clusters, but instead formed its own cluster between clusters 3 and 4 (Supplementary Figure 7) and was highly enriched in the small LD EVs. Interestingly, this protein has been suggested to be involved in membrane trafficking through the early endosomes [44–46], again linking the small LD EVs to the endosomal pathway.

Taken together, these results show that the vesicles isolated from the LD fractions – mainly small LD vesicles but also the large LD vesicles to some extent – are enriched in proteins associated with GO terms that suggest that these are the vesicles most similar to vesicles derived from the endosomal pathways, commonly called exosomes. In contrast, the two HD fractions clustered separately from each other. The small HD EVs clustered with the small EVs, and the proteins enriched in these two subpopulations were associated with the proteasome and nucleus, suggesting that these proteins are pelleted at 118,000 × *g*_avg_ but do not float to the lower densities in a density gradient.

### The presence of classical EV markers in tumour tissue-derived subpopulations of EVs

**Figure 8.**
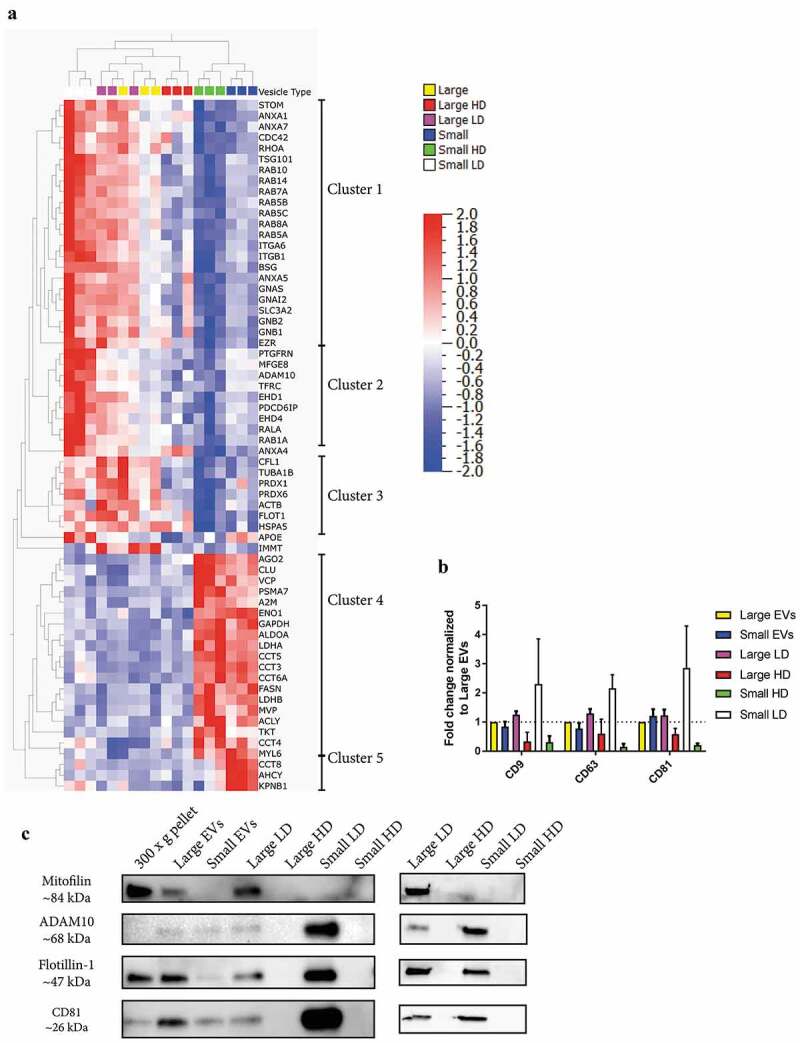
Presence of classical EV markers in EV subpopulations from metastatic melanoma tissues. The top 100 proteins identified in EVs from three online EV databases – EVpedia, ExoCarta and VesiclePedia [31–33] – were downloaded. Additionally, new markers suggested by Kowal and colleagues [6] as well as protein argonaute 2 were added to the list. After duplicates were removed the list contained 139 proteins. (a) A multi-group comparison was performed in Qlucore and showed that 64 of these proteins were differentially expressed in our dataset (*p*-value = 0.01, *q*-value = 0.02). **A2M**, alpha-2-macroglobulin; **ACTB**, actin; **ACYL**, ATP-citrate synthase; **ADAM10**, a disintegrin and metalloproteinase domain-containing protein 10; **AGO2**, protein argonaute 2; **ALDOA**, fructose-bisphosphate aldolase A; **ANX**, annexin; **APOE**, apolipoprotein E; **CCT**, T-complex protein; **CFL1**, cofilin-1; **EHD**, EH domain-containing protein; **EZR**, ezrin; **FASN**, fatty acid synthase; **FLOT1**, flotillin-1; **GAPDH**, glyceraldehyde 3-phosphate dehydrogenase; **GN**, guanine nucleotide-binding protein; **HSP**, heat shock protein; **IMMT**, mitofilin; **MFGE8**, lactadherin; **MVP**, major vault protein; **PDCD6IP**, programmed cell death 6-interacting protein; **PTGFRN**, prostaglandin F2 receptor negative regulator; **PRDX**, peroxiredoxin; **RAB**, Ras-related protein; **STOM**, erythrocyte band 7 integral membrane protein; **TRFC**, transferrin receptor protein 1; **VCP**, transitional endoplasmic reticulum ATPase. (b) Fold change of CD9, CD63 and CD81 in all subpopulations of EVs compared to large EVs, *N* = 3. (**c**) Western blot was performed to validate a selection of proteins from the TMT analysis. For the first Western blot, a total of 4 µg were loaded per sample. However, for the sample large HD EVs the yield was not enough and then 36 µl of EVs were loaded instead (0.6 µg of proteins). For the second Western blot, 23, 0.7, 25 and 2.5 µg was loaded for large LD, large HD, small LD and small HD, respectively. *N* = 2.

Next, we constructed a list of common EV-associated proteins (see methods). A multi-group comparison was performed and showed that 64 of these proteins were differentially expressed in our dataset (*p*-value = 0.01, *q*-value = 0.020877), and with an unsupervised hierarchical clustering these 64 proteins were separated into five clusters (Figure 8(a)). Cluster 1, which was mainly enriched in the two LD samples irrespective of size, contained proteins such as TSG101, ezrin, RAB proteins (RAB5A/B/C, RAB7A, RAB8A, RAB10, RAB14), annexins (ANX1, ANXA5, ANXA7), and G proteins (GNB1, GNB2, GNG12, GNAS). This suggests that these proteins are markers for vesicles with a density of approximately 1.11 g/cm^3^ but cannot be used to distinguish small and large vesicles.

Cluster 2 contained proteins that were mainly enriched in small LD EVs. Proteins in cluster 2 included EH domain-containing protein 1 and 4 (EHD4 and EHD1), prostaglandin F2 receptor negative regulator (PTGFRN), lactadherin, RAB1A, ADAM10, and programmed cell death 6-interacting protein (also called ALIX) (Figure 8(a)). Both ADAM10 and EHD4 have previously been suggested to be enriched in small LD EVs [6], and we could confirm this because they were also highly enriched in small LD EVs compared to all other samples in our dataset, including the large LD EVs. PTGFRN is a protein that has been shown to interact with two tetraspanins commonly identified in EV studies, CD9 and CD81 [47,48]. In our analysis, none of the three most common tetraspanins in EVs – CD9, CD63 and CD81 – were significantly different between the different EV isolates; however, the trend was that all three were most enriched in small LD EVs where PTGFRN was also enriched (Figure 8(b)).

Cluster 3 was enriched in the two LD EVs (small LD EVs and large LD EVs), as well as in large EVs (Figure 8(a)). This cluster contained flotillin-1, peroxiredoxin-1 and −6, cofilin-1, actin, and a heat shock protein (HSPA5). Although several of these proteins have been suggested to be markers for small EVs [49], their presence in both large and small EVs in our dataset shows that they cannot distinguish small and large EV subpopulations. Similar concerns have been raised previously for some of these proteins [6].

Clusters 4 and 5 were enriched in small EVs and small HD EVs (Figure 8(a)). These clusters contained several T-complex proteins (CCT3, CCT5, CCT6A and CCT8), ATP-citrate synthase, major vault protein, protein argonaute 2, GAPDH, alpha-2-macroglobulin, fatty acid synthase and transitional endoplasmic reticulum ATPase, suggesting that these clusters might be partly made up of particles or of vesicles with low lipid content and high protein and/or nucleic acid content or partly made up of protein complexes (density of approximately 1.16–1.19 g/cm^3^).

Annexin 4, apolipoprotein E and mitofilin had distinct expression profiles and did not end up in any of the clusters. Interestingly, mitofilin was the only protein enriched exclusively in large EVs and large LD EVs (Figure 8(a)), which supports previous findings [6].

Western Blot was used to validate a few of the proteins identified in the quantitative mass spectrometry analysis. One protein enriched in large EVs/large LD EVs (mitofilin), two protein enriched in small LD EVs (ADAM10 and CD81) and one protein enriched in both large LD and small LD EVs were chosen (Flotillin-1, Figure 8(c)). As expected mitofilin was enriched in the large EVs and large LD EVs as well as the 300 × *g* cell pellet and not detectable in the other samples and ADAM10 was enriched exclusively in small LD EVs, both validating the mass spectrometry analysis. Flotillin-1 and CD81 were observed to be enriched in both large LD and small LD EVs, indicating that these two markers cannot be used to separate large and small LD EVs (Figure 8(c)).

Together, these results show that several subpopulations of EVs can be isolated from melanoma tissue, but importantly they also support previous findings that several markers believed to be specific for small EVs are also enriched in large EVs.

## Discussion

Our results confirm that metastatic melanoma tissues contain a mixture of tumour cells and immune cells. Further, large quantities of vesicles of different sizes with a similar morphology as EVs can be found in the tumour interstitial space. Importantly, several subpopulations of EVs could be separated after isolation directly from the metastatic melanoma tissues using a protocol consisting of enzymatic treatment, differential ultracentrifugation and density gradient separation. These subpopulations of EVs were distinctly different in size and buoyant density, as well as in RNA and protein cargo.

First, we compared three isolation methods and found that the addition of collagenase D and DNase I to the tissue during EV release increased the solubility of the EV pellets, which consequently increased EV yield. Our results showing that EVs can be isolated directly from human melanoma tissues are in agreement with previous studies of EVs in brain and adipose tissue [19,21]. However, our work expands beyond the previous studies because our protocol allowed several subpopulations of EVs of different sizes and densities to be separated. Both at the protein and RNA level, it was clear that EVs were more similar and clustered based on density than on size. For RNA, the ribosomal RNA peaks could be seen with a Bioanalyzer® in both small and large LD EVs, but not in HD EVs (Figure 6(e)). Additionally, quantitative proteomic analysis also showed that the large and small EVs clustered based on density more than on size (Figure 7(a)). In our broad quantitative protein analysis, we observed that the LD EVs and particularly the small LD EVs were associated with the GO terms “Endosome”, “Plasma membrane” and “Extracellular exosome”, while the proteomes of the larger vesicles were more associated with the “Mitochondria” and “Endoplasmic reticulum” than the small EVs. We recently published a study identifying mitochondrial inner membrane proteins in EVs as a potential biomarker of metastatic melanoma [20]. In that study, we mixed large and small EVs on the same density cushions to explore the presence of potential candidates, but our current data separating on size suggest that this subpopulation of EVs is most likely associated with large EVs.

We could identify several previously suggested EV proteins that were enriched in individual EV subpopulations. Two such proteins – ADAM10 and EHD4 – were enriched in the small LD EVs, which confirmed recent findings in cell line-derived EVs [6]. Interestingly, ADAM10 which we could also confirm with Western blot, formed a small cluster within cluster 2 (Figure 8(a)) together with transferrin receptor protein 1, which was the protein studied when endosome-derived vesicles, later named exosomes, were discovered in the early 1980s [50,51]. This suggests that ADAM10 and the other proteins in this cluster might be part of endosomal-derived vesicles (also called exosomes).

The only protein enriched exclusively in large EVs and large LD EVs was the mitochondrial inner membrane protein mitofilin, located between clusters 3 and 4 in Figure 8(a). First, this confirms previous findings showing that mitofilin was only identified in large EVs isolated form cell lines [6]. Second, this shows that the large majority of the currently available EV markers do not describe large EVs well. This is not surprising, however, because the majority of proteomics studies so far have been conducted on the subpopulation of exosomes/small EVs. This highlights the need to perform proteomics studies that include several different subpopulations of EVs in order to determine both the shared and unique protein markers for these subpopulations.

We also identified several classical EV marker proteins that were not enriched in individual EV subpopulations but were instead present in several subpopulations. Our observations show that that flotillin-1 is present in several types of EVs, which supports previous findings [6], and also add proteins such as cofilin-1 and several RABs and annexins to the list of proteins that are not unique to small EVs. The presence of protein argonaute 2 in small EVs has been debated [35,52,53]; but in accordance with a recent study, we found protein argonaute 2 to be enriched in the small HD EVs [34].

One of the proteins enriched in the small EVs and small HD EVs was ATP-citrate synthase (Figure 8(a)), and interestingly, this was among the proteins that have recently been identified as enriched in so-called exomers, which are non-membranous nanoparticles approximately 35 nm in size [10]. When we further analysed our data, we could identify additionally enriched exomere-enriched proteins – UTP-glucose-1-phosphate uridylyltransferase and actin-related protein 3 [10] – in the small EVs and small HD EVs, suggesting that these fractions contain at least some exomers. Although we cannot determine to what degree exomers are present in our isolates, it is clear that these three proteins are present in our small HD EVs.

Three other proteins enriched in the small EVs and small HD EVs were GAPDH, fatty acid synthase and transitional endoplasmic reticulum ATPase. Interestingly, another recent paper also reported that these proteins are present in the small HD EVs [34]. However, they also suggested that enolase 1 and fructose-bisphosphate aldolase A are associated with small LD EVs, while in our data set these were also enriched in the small HD EVs.

In conclusion, we have established a novel method for isolating subpopulations of EVs directly from tumour tissues, which is important for several reasons. First, cell-line cultures lack the complexity of the tumour microenvironment where tumour cells, structural cells and immune cells are all interacting. Second, tumour tissue-derived EVs serve as a much more informative source compared to cell line-derived EVs because cell lines might not represent the tumour very well. Third, tumour EVs can be isolated from body fluids, but these will then be in a mixture together with EVs of other cellular and organ origin. By carefully isolating EVs directly from the tumour tissue, a snapshot of the EVs present in the tumour can be obtained. Because these EVs have the potential to later reach the circulation, a targeted analysis of the circulating EVs can be performed with this knowledge. We recently used this protocol and approach to identify EV-associated biomarker candidates in melanoma [20] demonstrating its potential use to successfully identify biomarkers candidates. Additionally, we have performed a comprehensive characterization of the isolated EV subpopulations and thus provide insight into the subpopulations of EVs resident in melanoma tumour tissue. We suggest that this method and findings can be used to improve biomarker discovery in cancers beyond just melanoma. Future studies may also explore the subpopulation of vesicles produced by different cells present in tumour tissues, such as inflammatory cells, fibroblasts or cancer cells.

## Supplementary Material

Supplemental Material
