# Supplementary material for: Subpopulations of extracellular vesicles from human metastatic melanoma tissue identified by quantitative proteomics after optimized isolation
Source: J Extracell Vesicles. 2020 Feb 11;9(1):1722433. doi: 10.1080/20013078.2020.1722433 (PMC7034452; doi:10.1080/20013078.2020.1722433)
Supplement: Supplemental Material [file ZJEV_A_1722433_SM3101.zip › Supplemenatry/Supplementary legends.docx]

**Supplementary Table 1**. Information and characteristics of the melanoma metastases used in this study and the experiments that were performed.

**Supplementary Figure 1. Four representative melanoma metastases**. (**A**) Cutaneous metastasis from the leg, (**B**) metastasis from the bowel, (**C**) metastasis from the liver, and (**D**) metastasis from a lymph node.

**Supplementary Figure 2. Schematic overview of the three different methods used to isolate vesicles from metastatic melanoma tissues**. Blue arrows indicate the procedures used for Protocol 1 (no collagenase D and no DNase I treatment). Green arrows indicate the procedures used in Protocol 2 (collagenase and DNase I treatment on vesicle-enriched pellets). Red arrows indicate the procedures used in Protocol 3 (collagenase D and DNase I treatment directly on melanoma pieces after chopping).

**Supplementary Figure 3. Characterization of large and small EVs isolated from metastatic melanoma tissues with Protocols 1 and 2**. (**A-B**) Large and small EVs isolated with Protocol 1 (**A**) and Protocol 2 (**B**) were visualized with transmission electron microscopy, and their RNA profiles were determined with a Bioanalyzer®. Scale bars = 200 nm. (**C-D**) Amount of vesicles (expressed in micrograms of proteins) isolated from large EVs and small EVs and normalized on weight of tumor (expressed in grams) for each protocol; protocol 1, N = 4; protocol 2, N = 2; protocol 3, N = 13.

**Supplementary Figure 4. Evaluation of the molecular effects of collagenase D and DNase I on CD9, CD63 and CD81. (A-B)** The CD9, CD63 and CD81 expression after collagenase D and DNase I treatment on HMC-1 cells **(A)** and on large and small EVs (bound to anti-CD63 beads) **(B)**, determine with Flow cytometry. HMC1 cell viability was tested using 7-ADD. N = 2. **(C)** ExoView^TM^ showing expression of CD9, CD63 and CD81 on total EVs in conditional media after enzymatic treatment with collagenase D and DNase I of HMC-1 cells. The results are presented as average ± SEM, N = 3.

**Supplementary Figure 5. Close up electron micrographs of subpopulations of tissue-derived EVs. (A-B)** Representative micrographs of large and small EVs **(A)** as well as large LD EVs, large HD EVs, small LD EVs and small HD EVs **(B)**. The scale bar is 100 nm. Large and small EVs; N = 8, large LD EVs, large HD EVs, small LD EVs and small HD EVs; N = 2.

**Supplementary Figure 6. Quantitative mass spectrometry of subpopulations of EVs**. (**A**) All six subpopulations of EVs were isolated from tumors from three different patients. All six samples from one tumor were run in the same TMT set resulting in three TMT sets. The layout of the three sets is shown. (**B**) The Venn diagram compares the proteins quantified in the three sets/tumors.

**Supplementary Figure 7. Multi-group comparison of the quantified proteins in EV subpopulations from metastatic melanoma tissues**. A multi-group comparison showed 742 proteins that were differentially expressed among the samples (p-value = 0.001, q-value = 0.009), and when an unsupervised hierarchical clustering was performed these proteins were divided into six large clusters.
