# Supplementary material for: Subpopulations of extracellular vesicles from human metastatic melanoma tissue identified by quantitative proteomics after optimized isolation
Source: J Extracell Vesicles. 2020 Feb 11;9(1):1722433. doi: 10.1080/20013078.2020.1722433 (PMC7034452; doi:10.1080/20013078.2020.1722433)
Supplement: Supplemental Material [file ZJEV_A_1722433_SM3101.zip › Supplemenatry/Supplementary Table 1.final.docx]

| **Supplementary Table 1 - samples information** | | | | | |
| --- | --- | --- | --- | --- | --- |
| **No** | **Type of melanoma** | **Type of metastasis** | **Stage** | **Labs** | |
| 1 | cutaneous melanoma | subcutaneus metastasis, breast | IV | protocol 1 and 2, RNA, EM, protein concentration | |
| 2 | cutaneous melanoma | in-transit metastasis, thigh | III | protocol 1, RNA, EM, protein concentration | |
| 3 | cutaneous melanoma | cutaneous metastasis, scalp | IV | protocol 1 and 2, RNA, protein concentration | |
| 4 | cutaneous melanoma | small bowel metastasis | IV | protocol 3, protein concentration | |
| 5 | cutaneous melanoma | lymph node metastasis, iliac | III | protocol 3, RNA, protein concentration | |
| 6 | cutaneous melanoma | lymph node metastasis, iliac | III | protocol 3, RNA, protein concentration | |
| 7 | cutaneous melanoma | small bowel metastasis | IV | protocol 3, RNA, EM, protein concentration | |
| 8 | cutaneous melanoma | lymph node metastasis, inguinal | III | protocol 3, RNA, EM, protein concentration | |
| 9 | uveal melanoma | liver metastasis | IV | protocol 3, RNA, protein concentration | |
| 10 a | cutaneous melanoma | in-transit metastasis, leg | III | protocol 3, RNA, EM, protein concentration | |
| 10 b | cutaneous melanoma | in-transit metastasis, leg | III | protocol 3, RNA, EM, protein concentration | |
| 10 c | cutaneous melanoma | in-transit metastasis, leg | III | protocol 1, RNA, protein concentration | |
| 11 | cutaneous melanoma | lymph node metastasis, axilla | III | IHC, EM sections (chemical fixation) | |
| 12 | cutaneous melanoma | lymph node metastasis, iliac | III | protocol 3, EM sections (chemical fixation) | |
| 13 | uveal melanoma | liver metastasis | IV | protocol 3, EM sections (high pressure freezing) | |
| 14 | cutaneous melanoma | small bowel metastasis | IV | protocol 3, EM, NTA, protein concentration, WB | |
| 15 | cutaneous melanoma | lymph node metastasis, axilla | III | protocol 3, NTA, protein concentration | |
| 16 a | cutaneous melanoma | lymph node metastasis, axilla | III | protocol 3, EM, NTA | |
| 16 b | cutaneous melanoma | in-transit metastasis, axilla | III | protocol 3, RNA, TMT, EM, NTA, protein concentration | |
| 17 | cutaneous melanoma | lymph node metastasis, axilla | III | protocol 3, RNA, TMT, EM, NTA, protein concentration | |
| 18 | cutaneous melanoma | lymph node metastasis, axilla | III | protocol 3, NTA | |
| 19 | cutaneous melanoma | primary nodular melanoma (pT4b), back | II | protocol 3, NTA | |
| 20 | cutaneous melanoma | in-transit metastasis, arm | III | protocol 3, RNA, TMT, EM, NTA, protein concentration | |
| 21 a | cutaneous melanoma | cutaneous metastasis, axillary region | IV | IHC, FC | |
| 21 b | cutaneous melanoma | cutaneous metastasis, lateral flank | IV | IHC, FC | |
| 21 c | cutaneous melanoma | subcutaneus metastasis, hypochondrium | IV | IHC, FC | |
| 22 a | cutaneous melanoma | in-transit metastasis, leg | III | IHC | |
| 22 b | cutaneous melanoma | lymph node metastasis, inguinal | III | IHC, cytospin, FC | |
| 23 | cutaneous melanoma | lymph node metastasis, axilla | III | cytospin, FC | |
| 24 a | cutaneous melanoma | in-transit metastasis, scapular region | III | cytospin, FC | |
| 24 b | cutaneous melanoma | in-transit metastasis, scapular region | III | cytospin, FC | |
| 24 c | cutaneous melanoma | in-transit metastasis, scapular region | III | cytospin, FC | |
| 25 | cutaneous melanoma | lymph node metastasis, axilla | III | WB | |
| 26 | cutaneous melanoma | in-transit metastasis, axillary region | III | WB | |
| 27 | cutaneous melanoma | in-transit metastasis, arm | III | WB | |
|  |  |  |  |  | |
| EM=electron microscopy; FC=flow cytometry; ICH=immunohystochemistry; NTA=nanoparticle tracking analysis; TMT=tandem mass tag; WB=western blot | | | | | |
| Tumors indicated with same number belong to the same patient | | | |  |  |
| Melanoma staging system according AJCC 8th edition. | | | |  |  |
